# Efficacy and External Validity of Electronic and Mobile Phone-Based Interventions Promoting Vegetable Intake in Young Adults: A Systematic Review Protocol

**DOI:** 10.2196/resprot.4665

**Published:** 2015-07-28

**Authors:** Monica Marina Nour, Juliana Chen, Margaret Allman-Farinelli

**Affiliations:** ^1^ Charles Perkins Centre School of Molecular Bioscience University of Sydney Sydney, NSW Australia

**Keywords:** young adults, vegetables, mHealth, eHealth, social marketing

## Abstract

**Background:**

Despite social marketing campaigns and behavior change interventions, young adults remain among the lowest consumers of vegetables. The digital era offers potential new avenues for both social marketing and individually tailored programs, through texting, web, and mobile applications. The effectiveness and generalizability of such programs have not been well documented.

**Objective:**

The aim of this systematic review is to evaluate the efficacy and external validity of social marketing, electronic, and mobile phone-based (mHealth) interventions aimed at increasing vegetable intake in young adults.

**Methods:**

The Preferred Reporting Items for Systematic Reviews and Meta-Analysis (PRISMA) protocol will be used to conduct this systematic review. The search strategy will be executed across eleven electronic databases using combinations of the following search terms: “online intervention”, “computer-assisted therapy”, “internet”, “website”, “cell phones”, “cyber”, “telemedicine”, “email”, “social marketing”, “social media”, “mass media”, “young adult”, and “fruit and vegetables”. The reference lists of included studies will also be searched for additional citations. Titles and abstracts will be screened against inclusion criteria and full texts of potentially eligible papers will be assessed by two independent reviewers. Data from eligible papers will be extracted. Quality and risk of bias will be assessed using the Effective Public Health Practice Project (EPHPP) Quality Assessment Tool for Quantitative Studies and The Cochrane Collaboration Risk of Bias assessment tool respectively. The external validity of the studies will be determined based on components such as reach, adoption, and representativeness of participants; intervention implementation and adaption; and program maintenance and institutionalization. Results will be reported quantitatively and qualitatively.

**Results:**

Our research is in progress. A draft of the systematic review is currently being produced for publication by the end of 2015.

**Conclusions:**

The review findings will assist the design and implementation of future eHealth and mHealth programs aimed at improving vegetable consumption in young adults.

**Trial Registration:**

PROSPERO International Prospective Register of Systematic Reviews: CRD42015017763; http://www.crd.york.ac.uk/PROSPERO/display_record.asp?ID=CRD42015017763#.VVKtqfmqqko (Archived by WebCite at http://www.webcitation.org/6YU2UYrTn).

## Introduction

### The Forgotten Age Group

Despite national and global social marketing campaigns and behavior change interventions, the current population’s intake of vegetables remains low [1]. Among Australian adults, young adults are least likely to meet the recommended five or more serves a day [2]. As they transition from parental supervision to independent living, young adults are establishing self-determined food habits that will have implications for their future health. It can take decades before diet-related diseases appear; however, a strong association has been established between fruit and vegetable consumption and a decreased risk of chronic diseases [3-11]. For this age group, promoting the well-established long-term health benefits of vegetable consumption, as is typically done in nationwide social marketing campaigns, is not a salient enough motivator for this population, who are typically unconcerned about their future health and engage in more high-risk behaviors [12-14]. This age group needs to be targeted separately in social marketing campaigns and behavior change interventions. Promoting the benefits they value, such as enhanced performance and physical ability, short-term health outcomes, and improved appearance may have greater impact.

### Digitalization of Interventions

The rise of the digital era offers potential new avenues for both social marketing and individually tailored programs, through texting, web and mobile apps to deliver health messages and facilitate change. Research indicates that electronic (eHealth) and mobile phone (mHealth)-based strategies are a promising channel for the delivery of interventions aimed at promoting healthful behaviors [15-17]. Young adults are among the most frequent users of these wireless information sharing platforms [18], and the total number of people using social networks is increasing rapidly [19]. Harnessing this technology could allow for the widespread dissemination of interventions in a low cost, accessible, convenient, and age-appropriate manner.

### Assessing Efficacy

When assessing the efficacy of interventions, the degree to which they effectively incorporate behavior change theories should be considered. A review of recent eHealth and mHealth interventions revealed that interventions which included more behavior change techniques had larger effects compared to those that used fewer techniques [20]. Furthermore, consideration of the accuracy of measurement of fruit and vegetable intake is crucial when evaluating the effectiveness of interventions. Fruits and vegetables have varying nutrient profiles and product attributes, and thus should be promoted separately. Additionally, the assessment of vegetable intake should be measured separately from fruit using validated tools.

### Assessing External Validity

Assessment of the external validity of studies is as equally important as determining efficacy. The external validity of studies has implications on the translation of interventions to the broader young adult population. With the young adult population neglected from many population-wide fruit and vegetable campaigns, investigation of the potential upscaling of current interventions is necessary.

To our knowledge, there is no published review to date focusing on the efficacy and generalizability of social marketing and eHealth and mHealth interventions on vegetable intake in young adults. This review addresses this gap in the literature.

Thus the aims are to: (1) systematically examine the effectiveness of social marketing, electronic and/or mobile phone-based interventions in increasing fruit and vegetable intake in young adults; (2) assess the efficacy/validity of tools used to monitor changes in fruit and vegetable intake; and (3) review the adequacy of reporting of external validity components.

## Methods

### Defining Search Terms

The Preferred Reporting Items for Systematic Reviews and Meta-Analysis (PRISMA) protocol will be used to conduct this systematic review [21]. The search terms have been selected to be broad and will include combinations, truncations, and synonyms of “online intervention”, “computer-assisted therapy”, “Internet”, “website”, “cell phones”, “cyber”, “telemedicine”, “email”, “fruit and vegetables”, “young adult”, and “randomized controlled trials”. A separate search will be conducted to identify studies reporting interventions using social marketing and mass media to increase fruit and vegetable intake in young adults. This search will encompass terms such as “young adult”, “fruit and vegetables”, “social marketing”, “social media”, and “mass media”. The Medline thesaurus Medical Subject Headings (MESH) terms will be refined according to each database. Although we are primarily interested in the implications of interventions on vegetable intake, the search term was broadened to include “fruit” as studies commonly report on fruit and vegetables concurrently.

### Search Strategy

The following electronic databases will be searched for papers published between January 1990 and March 2015: the Cochrane Library, Cochrane Library of Systematic Reviews, Cochrane Central Register of Controlled Trials, CINAHL, Medline, Embase, PubMed, PyschINFO, Scopus, Web of Science, and Science Direct. The start of 1990 was selected, as it corresponded with the period during which the use of email became widespread [22]. Reference lists and JMIR journals will be hand searched for additional citations. Studies determined to be relevant to the review will be included.

### Eligibility Criteria

#### Overview

The eligibility criteria for studies have been selected based on participants, interventions, comparisons, outcomes, and study designs (PICOS). Only studies written in English and published after 1990 will be included.

#### Participants

The target age group for the included studies will be young adults aged 18-35 years inclusive. The participants should be healthy, with no disease or illness which would impact the primary outcome or ability to modify fruit and vegetable intake. There will be no limitation based on gender, ethnicity or socioeconomic status. Interventions set outside of universities will also be included in the review.

#### Interventions

The type of interventions that will be considered in the initial search will be eHealth or mHealth-based interventions. These are studies that employ the use of mobile phone apps, texting, email, phone calls, and websites to deliver the intervention. The secondary search will not be limited to eHealth or mHealth interventions and will include social marketing and mass media interventions. These are defined as studies that employ the use of media advertising through television, radio, billboards, and/or social media platforms as well as other community-based activities such as group education and cooking classes to increase fruit and vegetable intake.

#### Comparisons

Comparisons will be made between baseline and follow up results within and between studies. The differences between intervention and control arms (no intervention or minimal contact) will also be explored.

#### Outcomes

The primary outcome that will be investigated is the change in fruit and vegetable intake between baseline and follow-up. This can be reported in serves, frequency or grams. Fruit will be included as an outcome to account for studies reporting fruit and vegetable intake concurrently.

#### Study Designs

The first search will be limited to randomized controlled trials (RCTs) or cluster-RCTs with an aim of increasing fruit and vegetable intake in young adults. The social marketing search will not be limited by study design.

### Study Selection

**Figure 1:**
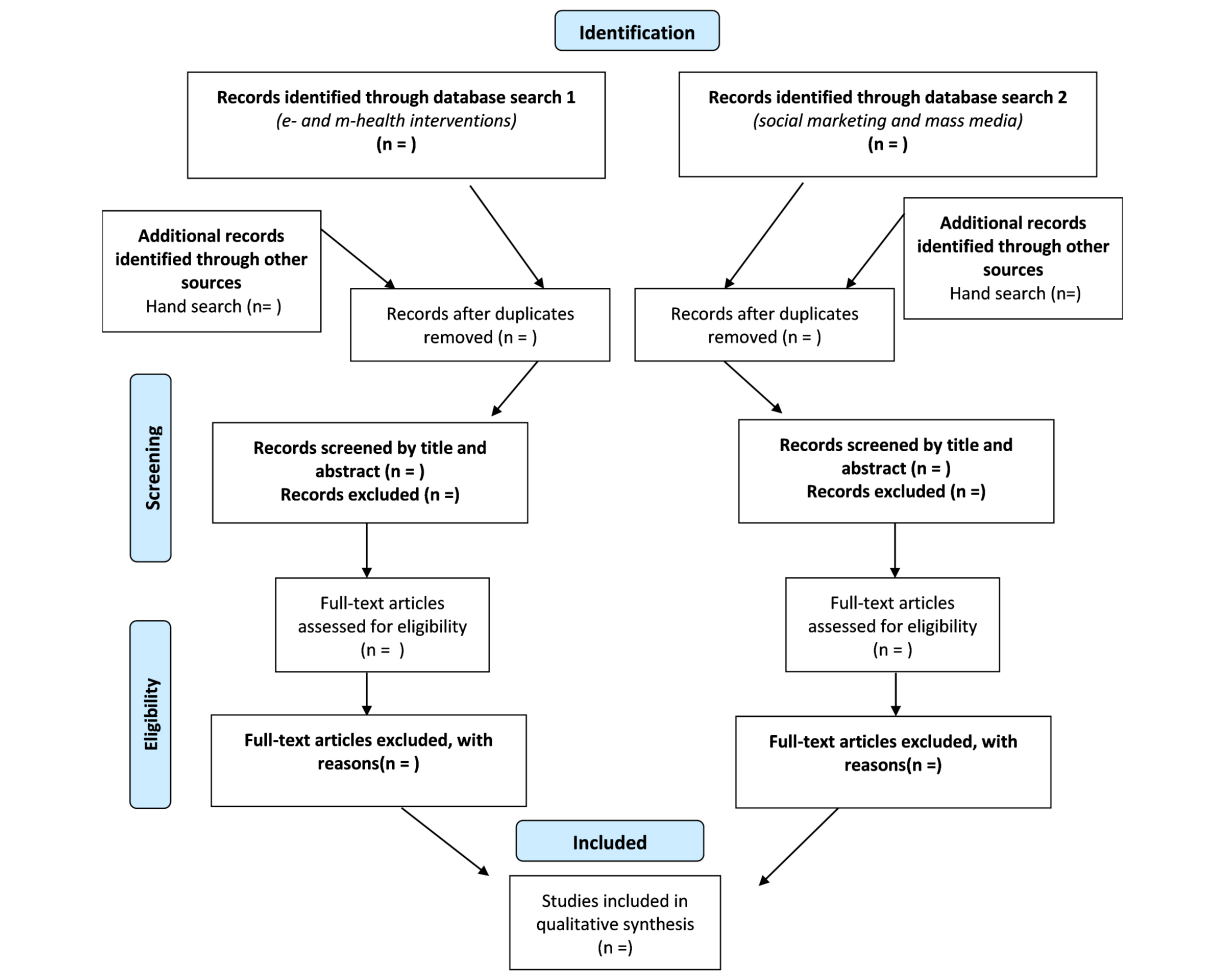
Preferred reporting items for systematic reviews and meta-analyses (PRISMA) flow diagram.

Titles and abstracts of all retrieved studies will be exported to Endnote X6 citation management software (Thomson Reuters, Philadelphia, PA, USA). Duplicates will be deleted before titles and abstracts are reviewed to group papers into either of the following: (1) meeting selection criteria; (2) requiring further examination; or (3) excluded. Papers determined as potentially relevant to the review will be downloaded as full text and reviewed for eligibility by two evaluators (MMN, JC) and further categorized (Figure 1). Discrepancies in evaluators’ results will be resolved by discussion and, when necessary, in consultation with a third reviewer (MAF). The reasons for exclusion of studies will be recorded in a PRISMA flowchart which will illustrate the search, screening, and selection results (Figure 1).

### Data Collection

A data extraction table will be designed using principles of the PRISMA statement for reporting systematic review [21], and the Cochrane Collaboration’s tool for assessing risk of bias [23]. Once piloted for use on included studies, the following data will be collected: study details (authors, year, country of publication, funding, and affiliations); participants (characteristics, setting, inclusion/exclusion criteria, attrition, and blinding); intervention and comparator details; duration; and outcome measure (change in fruit and vegetable intake).

### Data Analysis

#### Reporting of Intervention Outcomes

An appropriate method of reporting the treatment effect will be determined based on the type of data extracted from included studies. It is anticipated that the mean differences in fruit and vegetable intake between baseline and follow up will be reported. These results will be tabulated to enable qualitative description of results and heterogeneity assessment for potential pooling of results using meta-analysis.

#### Risk of Bias Assessment

Using the Cochrane Collaboration's tool [23], risk of bias will be determined for each included study, taking into consideration selection (random sequence generation and concealment of allocation methods), attrition (completeness of outcome data), detection (blinding of participants and personnel), and reporting (selective reporting of outcome measures). Two authors (MMN and JC) will independently evaluate each study for risk of bias and will code them as low-risk, high-risk or unclear risk. Any discrepancies will be settled through discussion.

#### Quality Assessment

The quality of each study will be determined by two independent parties using the Effective Public Health Practice Project (EPHPP) Quality Assessment Tool for Quantitative Studies [24]. The following components will be considered in order to assign a quality rating to each study: study design, selection bias, blinding, confounders, outcome collection methods, participant withdrawals, and dropouts. Studies will be given a rating of “weak”, “moderate” or “strong” by two authors (MMN, JC), with conflicting ratings resolved through discussion with a third independent reviewer (MAF).

#### Rating External Validity

A table collating the reported external validity components of the included studies was designed based on the criteria for rating external validity developed by Green and colleagues [25]. The table explores components under three sections: (1) reach, adoption and representativeness of participants; (2) intervention implementation and adaption; and (3) program maintenance and institutionalization (sustainability of program implementation). Qualitative and quantitative data relating to these external validity components will be extracted. Extracted data will be used to report the number and percentage of studies adhering to the external validity components. The adequacy and frequency of reporting of these components will be explored between studies.

## Results

Our research is in progress. A draft of the systematic review is currently underway and will be submitted before the end of 2015.

## Discussion

This review will present a summary of the efficacy and external validity of the published studies that have used eHealth and mHealth or social marketing strategies to engage young adults in improving their vegetable intake. The findings will provide a scope for the development of future interventions and social marketing campaigns targeted at this age group.

## Appendix

Multimedia Appendix 1 MEDLINE Search Strategy.

## Abbreviations

EPHPP: Effective Public Health Practice Project
MESH: Medline thesaurus Medical Subject Headings
PICOS: participants, interventions, comparisons, outcomes and study designs
PRISMA: Preferred Reporting Items for Systematic Reviews and Meta-Analysis

## References

[1] Rekhy R, McConchie R (2014). Promoting consumption of fruit and vegetables for better health. Have campaigns delivered on the goals?. Appetite.

[2] (2012). Australian Health Survey: First Results, 2011–12.

[3] Ledoux TA, Hingle MD, Baranowski T (2011). Relationship of fruit and vegetable intake with adiposity: a systematic review. Obes Rev.

[4] Boffetta P, Couto E, Wichmann J, Ferrari P, Trichopoulos D, Bueno-de-Mesquita HB, Büchner FL, Key T, Boeing H, Nöthlings U, Linseisen J, Gonzalez CA, Overvad K, Tjønneland A, Olsen A, Clavel-Chapelon F, Boutron-Ruault M, Morois S, Lagiou P, Naska A, Benetou V, Kaaks R, Rohrmann S, Panico S, Sieri S, Vineis P, Palli D, Peeters PH, Lund E, Brustad M, Engeset D, Huerta JM, Rodríguez L, Sánchez M, Dorronsoro M, Barricarte A, Hallmans G, Johansson I, Manjer J, Sonestedt E, Allen NE, Bingham S, Khaw K, Slimani N, Jenab M, Mouw T, Norat T, Riboli E, Trichopoulou A, van Duijnhoven Fränzel J B, Nielsen Michael R S, van Gils Carla H (2010). Fruit and vegetable intake and overall cancer risk in the European Prospective Investigation into Cancer and Nutrition (EPIC). J Natl Cancer Inst.

[5] Lunet N, Lacerda-Vieira A, Barros H (2005). Fruit and vegetables consumption and gastric cancer: a systematic review and meta-analysis of cohort studies. Nutr Cancer.

[6] Steinmetz KA, Potter JD (1996). Vegetables, fruit, and cancer prevention: a review. J Am Diet Assoc.

[7] Dauchet L, Amouyel P, Hercberg S, Dallongeville J (2006). Fruit and vegetable consumption and risk of coronary heart disease: a meta-analysis of cohort studies. J Nutr.

[8] Ness AR, Powles JW (1997). Fruit and vegetables, and cardiovascular disease: a review. Int J Epidemiol.

[9] Appel LJ, Moore TJ, Obarzanek E, Vollmer WM, Svetkey LP, Sacks FM, Bray GA, Vogt TM, Cutler JA, Windhauser MM, Lin PH, Karanja N (1997). A clinical trial of the effects of dietary patterns on blood pressure. DASH Collaborative Research Group. N Engl J Med.

[10] Miura K, Greenland P, Stamler J, Liu K, Daviglus ML, Nakagawa H (2004). Relation of vegetable, fruit, and meat intake to 7-year blood pressure change in middle-aged men: the Chicago Western Electric Study. Am J Epidemiol.

[11] Bazzano LA, He J, Ogden LG, Loria CM, Vupputuri S, Myers L, Whelton PK (2002). Fruit and vegetable intake and risk of cardiovascular disease in US adults: the first National Health and Nutrition Examination Survey Epidemiologic Follow-up Study. Am J Clin Nutr.

[12] Dumbrell S, Mathai D (2008). Getting young men to eat more fruit and vegetables: a qualitative investigation. Health Promot J Austr.

[13] Mendis K, Forster T, Paxton K, Hyland K, Yelverton J, McLean R, Canalese J, Brown A, Steinbeck K (2014). Large and forgotten in rural Australia: assessment, attitudes and possible approaches to losing weight in young adult males. BMC Public Health.

[14] Benyamina A, Blecha L, Reynaud M (2012). Global burden of disease in young people aged 10-24 years. Lancet.

[15] Fjeldsoe BS, Marshall AL, Miller YD (2009). Behavior change interventions delivered by mobile telephone short-message service. Am J Prev Med.

[16] Free C, Phillips G, Galli L, Watson L, Felix L, Edwards P, Patel V, Haines A (2013). The effectiveness of mobile-health technology-based health behaviour change or disease management interventions for health care consumers: a systematic review. PLoS Med.

[17] Hurling R, Catt M, Boni MD, Fairley BW, Hurst T, Murray P, Richardson A, Sodhi JS (2007). Using internet and mobile phone technology to deliver an automated physical activity program: randomized controlled trial. J Med Internet Res.

[18] Rainie L (2012). Pew Internet.

[19] Chou WS, Hunt YM, Beckjord EB, Moser RP, Hesse BW (2009). Social media use in the United States: implications for health communication. J Med Internet Res.

[20] Webb TL, Joseph J, Yardley L, Michie S (2010). Using the internet to promote health behavior change: a systematic review and meta-analysis of the impact of theoretical basis, use of behavior change techniques, and mode of delivery on efficacy. J Med Internet Res.

[21] Moher D, Liberati A, Tetzlaff J, Altman DG (2010). Preferred reporting items for systematic reviews and meta-analyses: the PRISMA statement. Int J Surg.

[22] Moschovitis C, Poole H, Senft T (1999). History of the Internet: A Chronology, 1843 to the Present.

[23] Altman Douglas G, Gøtzsche Peter C, Jüni Peter, Moher David, Oxman Andrew D, Savovic Jelena, Schulz Kenneth F, Weeks Laura, Higgins Julian P T, Sterne Jonathan A C, Cochrane Bias Methods Group, Cochrane Statistical Methods Group (2011). The Cochrane Collaboration's tool for assessing risk of bias in randomised trials. BMJ.

[24] Thomas BH, Ciliska D, Dobbins M, Micucci S (2004). A process for systematically reviewing the literature: providing the research evidence for public health nursing interventions. Worldviews Evid Based Nurs.

[25] Green Lawrence W, Glasgow Russell E (2006). Evaluating the relevance, generalization, and applicability of research: issues in external validation and translation methodology. Eval Health Prof.

