# Supplementary material for: Efficacy and External Validity of Electronic and Mobile Phone-Based Interventions Promoting Vegetable Intake in Young Adults: A Systematic Review Protocol
Source: JMIR Res Protoc. 2015 Jul 28;4(3):e92. doi: 10.2196/resprot.4665 (PMC4705367; doi:10.2196/resprot.4665)
Supplement: Multimedia Appendix 1 [file resprot_v4i3e92_app1.pdf]

**Table 1: Electronic Database search: Medline (Search 1: e- and m-health interventions)**

| Search ID number | Search terms |
|---|---|
| 1 | Computer-assisted therapy.mp or Therapy, Computer-Assisted/ |
| 2 | Internet/ or Website.mp |
| 3 | Cell phones.mp or Cell phones/ |
| 4 | Telemedicine/ or Cyber.mp |
| 5 | email.mp or Electronic mail/ |
| 6 | Adult/or young adult/ or young adult*.mp |
| 7 | Fruit/ or Fruit*.mp |
| 8 | Vegetable*.mp or Vegetables/ |
| 9 | 1 or 2 or 3 or 4 or 5 |
| 10 | 7 or 8 |
| 11 | 6 and 9 and 10 |
| 12 | Limit 11 to (English language and humans and yr = 1990-current) |

**Table 2: Electronic Database search: Medline (Search 2: social marketing and mass media interventions)**

| Search ID number | Search terms |
|---|---|
| 1 | Adult/ or young adult/ or young adult*.mp. |
| 2 | Fruit/ or fruit*.mp. |
| 3 | Vegetable*.mp. or Vegetables/ |
| 4 | 2 or 3 |
| 5 | Social marketing.mp. or social marketing/ |
| 6 | Social media. mp or Mass Media/ or Social Media/ |
| 7 | 5 or 6 |
| 8 | 1 and 4 and 7 |
| 9 | Limit 11 to (English language and humans and yr = 1990-current) |
